# Radiosensitization of Rare-Earth Nanoparticles Based on the Consistency Between Its K-Edge and the X-Ray Bremsstrahlung Peak

**DOI:** 10.3390/jfb16020041

**Published:** 2025-01-24

**Authors:** Xiang Zhu, Cheng-Jie Qiu, Jiao-Jiao Cao, Dida Duosiken, Yuhan Zhang, Ben-Gen Pei, Ke Tao, Si-Jian Pan

**Affiliations:** 1 Department of Neurosurgery, Ruijin Hospital, School of Medicine, Shanghai Jiao Tong University, Shanghai 200020, China; zhusurg@163.com (X.Z.); qcj12352@rjh.com.cn (C.-J.Q.); cjj40997@rjh.com.cn (J.-J.C.); peibengen@163.com (B.-G.P.); 2 Department of General Surgery, Xinhua Hospital, School of Medicine, Shanghai Jiao Tong University, Shanghai 200092, China; 3 State Key Lab of Metal Matrix Composites, School of Materials Science and Engineering, Shanghai Jiao Tong University, Shanghai 200240, China; dida.d@sjtu.edu.cn (D.D.); zhangyuhan2003@sjtu.edu.cn (Y.Z.); 4 Department of Neurosurgery, Zhoupu Hospital, Shanghai University of Medicine & Health Sciences, Shanghai 201318, China

**Keywords:** radiosensitization, K-edge effect, Bremsstrahlung peak, thulium

## Abstract

Nanoparticle-based X-ray radiosensitization strategies have garnered significant attention in recent years. However, the underlying mechanisms of radiosensitization remain incompletely understood. In this work, we explore the influence of the K-edge effect in the X-ray absorption of nanomaterials on sensitization. Due to the alignment of the K-edge of thulium (Tm) with the Bremsstrahlung peak in the energy spectrum of medical X-ray accelerators, the following four different rare-earth nanomaterials with varying Tm percentages were designed: NaTmF_4_, NaTm_0.6_Lu_0.4_F_4_, NaTm_0.4_Lu_0.6_F_4_, and NaLuF_4_. We evaluated the X-ray absorption and the ability to generate secondary electrons and reactive oxygen species (ROS) of these nanoparticles. The radiosensitizing effect was evaluated through clonogenic assays. Our results showed that the K-edge effect affected secondary electron generation but did not significantly change ROS production. Nonetheless, NaTmF_4_ induced marginally more DNA damage in the U87 cells than the other cell types. NaTmF_4_ also exhibited superior radiosensitization efficacy against the U87 tumor cells. This shows that secondary electrons and ROS play pivotal roles in radiosensitization, which might be crucial to improving cancer treatment efficacy through enhanced radiation therapy outcomes.

## 1. Introduction

Radiotherapy (RT) has long been one of the primary modalities in cancer treatment [1,2]. However, the efficacy of conventional RT is often constrained by collateral damage to healthy tissues, limiting both the dosage and therapeutic outcomes. In recent years, nanoparticle-based radiosensitization has emerged as a promising and innovative strategy [3,4,5]. Due to their unique physicochemical properties and small size, nanoparticles can selectively accumulate in tumor sites and enhance the effects of radiation through radiosensitization [6,7]. By mechanisms such as increased radiation energy deposition, promotion of reactive oxygen species (ROS) production, and interference with tumor cell DNA repair, nanoparticles significantly enhance the effectiveness of RT [8,9]. This approach reduces the required radiation dose and improves treatment precision and efficiency, minimizing damage to the surrounding healthy tissues [10]. Consequently, nanoparticle-based radiosensitization has become a key focus in cancer treatment research, holding significant clinical potential [11,12].

Studies on the mechanism of radiosensitization concluded that the interaction of X-rays with sensitizing materials mainly generates secondary electrons, including photoelectrons, Auger electrons, Compton electrons, etc., which damage the cellular DNA and thus increase the damage of X-rays to tumor tissues [13,14,15]. It has also been reported that some secondary electrons interact with surrounding water and other substances to generate reactive oxygen species (ROS) with damaging ability [9,16]. The classical theory of mass attenuation coefficient discovered that the yield of secondary electrons is directly proportional to the fourth power of its atomic number (Z). This means that substances with a higher Z should have a stronger radiosensitizing effect [17,18]. For example, materials such as gold (Au, Z = 79), hafnium (Hf, Z = 72), gadolinium (Gd, Z = 64), platinum (Pt, Z = 78), and bismuth (Bi, Z = 83) have demonstrated their effectiveness in radiosensitizing therapies by accumulating and attenuating radiation energy [19,20,21,22,23,24]. However, nano diamonds with an atomic number of only 12 also exhibited a radiotherapy effect similar to gold nanoparticles [25,26]. In addition, the sensitization of nanomaterials predicted by Monte Carlo simulation calculations (MCNP) is often inconsistent with their experimental radiotherapy sensitization effects [27,28,29]. These observations challenged the classical consideration of radiosensitizer design, indicating that our understanding of nanoparticulate radiosensitizers remains limited.

It is believed that the dominant effect of nanoparticle-based X-ray radiosensitization is the photoelectric effect when keV X-ray energy is used. One crucial factor that affects the photoelectric effect is the K-edge. This occurs when the photon energy of the X-ray is close or equal to the K-edge orbital binding energy of the elements [30,31,32]. In such cases, the efficiency of the photoelectric effect can increase by several orders of magnitude. Yokoya et al. proved that more secondary electrons were produced when the X-ray photon energy was equal to the K-edge binding energy (2153 eV) of the phosphorus element than employing X-ray with other energy [33]. Although the K-edge energy of phosphorus can hardly be utilized in radiosensitization, this result indicated that the number of secondary electrons would be pronouncedly enhanced by matching the X-ray energy and K-edge of the elements.

**Figure 1:**
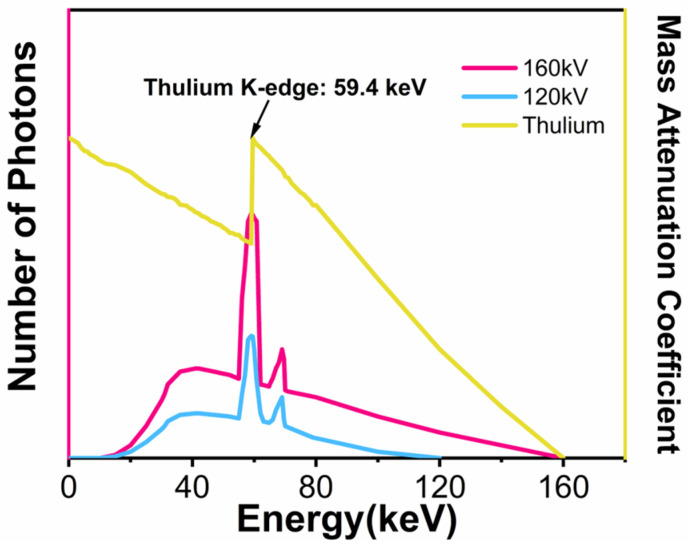
Schematic illustration of K-edge binding energy of thulium and Bremsstrahlung peak of clinical X-ray sources.

We noted that a Bremsstrahlung peak exists in all clinical X-ray sources at 59 keV [34]. This peak does not change with the voltage of the X-ray tubes, as illustrated in Figure 1. In the meantime, the element of thulium (Tm, Z = 69) has a K-edge energy of 59.4 keV [35], matching the Bremsstrahlung peak well. Therefore, we hypothesize that nanoparticles containing Tm might considerably contribute to radiosensitization. Consequently, we adjusted the percentage of Tm in NaLnF_4_ (Ln = lanthanides) nanoparticles to evaluate the influence of K-edge on radiosensitization. Specifically, NaTmF_4_, NaTm_0.6_Lu_0.4_F_4_, NaTm_0.4_Lu_0.6_F_4_, and NaLuF_4_ nanoparticles were prepared with the same size and morphology. Lutecium (Lu) was used as the control in these nanoparticles as it has a higher Z (71) than Tm and has a different K-edge (63.3 keV). We evaluated the X-ray attenuation, the generation of secondary electrons, and the reactive oxygen species (ROS) produced by these nanomaterials under X-ray irradiation and within cells. We also analyzed the effects of ROS and secondary electrons on the radiosensitization efficacy through clonogenic assays. Our work may suggest that the K-edge effect and secondary electron production contribute to improved radiosensitization, which might be meaningful for developing effective nanoparticulate radiosensitizers [36].

## 2. Materials and Methods

### 2.1. Materials

Aminophenyl fluorescein (APF) was obtained from Shanghai Maokang Biotechnology Co., Ltd., (≥95%); Dimethyl sulfoxide (DMSO) was sourced from Sinopharm Chemical Reagent Co., Ltd., (>99%) (Shanghai, China); Dulbecco’s Modified Eagle Medium (DMEM) and Penicillin/Streptomycin were purchased from Gibco, Waltham, MA, USA; Anti-phospho-H2AX (serine 139, γ-H2AX) antibody was obtained from Cell Signaling Technology, Danvers, MA, USA; 2′,7′-Dichlorodihydrofluorescein diacetate (DCFH-DA) and Mouse anti-β-Actin antibody were acquired from Shanghai Beyotime Biotechnology Co., Ltd., Shanghai, China; and Cell Counting Kit-8 (CCK-8) was sourced from Shanghai Jiayuan Biotechnology Co., Ltd., Shanghai, China.

### 2.2. Synthesis of NaTm_x_Lu_(1−x)_F_4_

The four NaTm_x_Lu_(1−x)_F_4_ nanoparticles (x = 1, 0.6, 0.4, 0), specifically NaTmF_4_, NaTm_0.6_Lu_0.4_F_4_, NaTm_0.4_Lu_0.6_F_4_, and NaLuF_4_, were synthesized using the thermolysis method, referencing the detailed preparation approach described by Qian et al. [37]. Initially, 2 mmol of CF_3_COONa was weighed and combined separately with 1 mmol of (CF_3_COO)_3_Tm, 0.6 mmol of (CF_3_COO)_3_Tm and 0.4 mmol of (CF_3_COO)_3_Lu, 0.4 mmol of (CF_3_COO)_3_Tm and 0.6 mmol of (CF_3_COO)_3_Lu, and finally, 1 mmol of (CF_3_COO)_3_Lu. These mixtures were then added to a 40 mL solution consisting of oleic acid (OA) and oleylamine (OM), maintaining a volume ratio of 10% OA to 90% OM. The reaction was conducted under vacuum conditions and heated to 110 °C, with continuous stirring for approximately 1 h. Under the protection of nitrogen gas, the temperature was rapidly increased to 330 °C and maintained for another hour with stirring. Afterward, the heating mantle was removed, and the mixture was allowed to cool to room temperature under a nitrogen atmosphere. The particles were then washed 2–3 times using a mixture of ethanol and cyclohexane, followed by centrifugation at 12,000 rpm for 5 min. Finally, the prepared nanoparticles were collected and dispersed in cyclohexane.

### 2.3. Surface Modification of NaTm_x_Lu_(1−x)_F_4_ with Silica

To prepare the samples, 10 mg of each synthesized nanoparticle type—NaTmF_4_, NaTm_0.6_Lu_0.4_F_4_, NaTm_0.4_Lu_0.6_F_4_, and NaLuF_4_—was separately dispersed in 15 mL of cyclohexane. To this dispersion, 0.5 mL of Igepal CO-520 and 80 μL of ammonia solution were added, followed by ultrasonication for 10 min. Subsequently, 20 μL of 3-Aminopropyl triethoxysilane (APTS) was introduced into the above emulsion under room temperature stirring conditions. After stirring for 48 h at room temperature, the nanoparticles were collected by centrifugation (12,000 rpm, 6 min) and washed three times with ethanol. Finally, the nanoparticles were dissolved in ultrapure water at a concentration of 1 mg/mL for storage.

### 2.4. TEM Microscope, EDS, XRD

Morphological characterization: The solution of the nanoparticles to be tested was diluted to approximately 1:100. Then, 10–20 μL was dropped onto a carbon film. After the carbon film had dried, it was observed using a 120 kV transmission electron microscope (TEM, Tecnai G2 Spirit Biotwin, FEI Company, Hillsboro, OR, USA) to obtain TEM images or a field emission transmission electron microscope (Talos F200X, Thermo-fisher Company, USA) to obtain high-resolution TEM (HRTEM) images and elemental distribution analysis (EDS) images.

Crystalline characterization: The nanoparticle powder to be tested was placed in an X-ray diffractometer (AXS D8 ADVANCE Da Vinci, Bruker Company, Billerica, MA, USA) to test its XRD spectrum.

### 2.5. CT Testing

Various gradient concentrations of the four nanoparticles were embedded in agarose gel and tested in 4 mL centrifuge tubes. The X-ray Computed Tomography (CT) scans were conducted using a Somatom Sensation 4 CT system (Siemens, Munich, Germany), with settings at 140 kVp, 50 mA, a slice thickness of 1.0 mm, and a scan duration of 5.3 s. The images and Hounsfield Unit (HU) values were measured and analyzed using the Siemens Inveon MMCT system.

### 2.6. Monte Carlo N Particle Transport Code (MCNP)

The MCNP simulation was performed using the Geant4 Monte Carlo toolkit (version 4.10.04). The specific settings for the calculation were as follows: The number of incident photons was set to 10 million, with energies of 59.4 keV and 63.3 keV. The incident width and the nanoparticle diameter were both configured to 10 nm. The density of the nanoparticles was set at 5.8 g/cm^3^. Finally, the total number of secondary electrons (including photoelectrons, Auger electrons, Compton scatter electrons) generated within a 200 μm radius around the nanoparticle was calculated.

### 2.7. Cell Culture and Cytotoxicity Test

Human glioblastoma cells U87, procured from ATCC (HTB-14™), were cultured in Dulbecco’s Modified Eagle Medium (DMEM) supplemented with 10% fetal bovine serum (FBS) and 1% penicillin/streptomycin. The cells were incubated in a culture incubator with the conditions maintained at 5% CO_2_, 90% relative humidity, and a temperature of 37 °C.

Cell cytotoxicity was measured using the Cell Counting Kit-8 (CCK-8). The U87 cells were seeded in a 96-well plate at a density of 1 × 10^4^ cells per well. After cell adhesion, they were co-cultured with various concentrations of nanoparticles (0, 50 μg/mL, 100 μg/mL, 200 μg/mL, 300 μg/mL, and 400 μg/mL) for 24 h. Subsequently, the cells were washed three times with PBS to remove free nanoparticles, followed by the addition of CCK-8 reagent to each well. After 4 h, the absorbance at 450 nm was read using a microplate reader (Thermo Fisher Scientific, Waltham, MA, USA). The experimental measurement of nanoparticles co-cultured with cells was designated as reading A; the reading for the medium and nanoparticle group as the conditional control was designated as reading B. Cells untreated with nanoparticles were set as the negative control and designated as reading C. The percentage of cell viability was calculated using the following formula: Cell Viability (%) = ((A − B)/(C − B)) × 100%

### 2.8. Cellular Uptake

In 6 cm culture dishes, the U87 cells adherent to the dish were exposed to various nanoparticles dissolved in the culture medium at a concentration of 50 μg/mL. After 4 h, the cells were washed twice with PBS and then digested with trypsin. Subsequently, cell counting was performed using an automatic cell counter (Thermo Fisher Scientific, Waltham, MA, USA). Following this, the levels of rare-earth elements (Tm or Lu) absorbed by the cells were quantified using Inductively Coupled Plasma Atomic Emission Spectroscopy (ICP-AES, iCAP 6000 Radial, Thermo Fisher Scientific, Waltham, MA, USA).

### 2.9. Colony Formation Assay

After culturing the U87 cells for 24 h, the original culture medium was discarded, and the cells were digested with trypsin, counted, and then suspended. The cells were divided into several groups, each with five parallel sets. After adding different materials (10 ug/mL), the cells were cultured in an incubator for 4 h. Subsequently, they were irradiated with different doses of radiotherapy (0, 1, 2, 3, and 4 Gy, at a dose rate of 160 kV, 25 mA, and 1.2 Gy/min). The cells were then plated in 6-well plates with 300 cells/well, thoroughly mixed and incubated in a CO_2_ incubator for 14 days. Once visible cell colonies were observed, the supernatant was discarded, and the cells were washed twice with PBS. The cells were then fixed with 4% paraformaldehyde at room temperature for 20 min, followed by another two PBS washes. The cells were stained with 0.5% crystal violet at room temperature for 20 min, and the excess crystal violet was gently washed off with PBS. After drying at room temperature, the colonies were observed and photographed. Colonies containing at least 50 cells were counted as a single colony formation. The seeding efficiency, survival rate, and sensitization enhancement ratio (SER) were calculated using the following formulas:

$$Seeding\;Efficiency=\frac{Number\;of\;Surviving\;Colonies}{Number\;of\;Cells\;Seeded}$$

$$Survival\;Rate=\frac{Number\;of\;Colonies\;Surviving\;Post\;Irradiation}{Number\;of\;Cells\;Seeded\times Seeding\;Efficiency}$$

*SER* describes the ratio of X-ray doses required to kill 63% or 90% of the total cells with and without sensitizing materials and is calculated as SER_D0_ or SER_D10_, respectively. It is computed using the following formula:

$$SER=\frac{D_{Control}}{D_{Experimental\;Group}}$$

where *D* is the radiation dose corresponding to a specific survival fraction (*SF*). The survival fraction is fitted to the following equation:

$$SF=1-(1-e^{-D/D0})$$

### 2.10. Western Blot (WB)

Proteins in the cell lysis solution can be directly used for γ-H2AX detection, and the cell extracts are prepared in an appropriate lysis buffer (50 mM Tris-HCl, pH 7.4, 150 mM NaCl, and 1% Nonidet P-40). Each sample is prepared with a sample buffer and then separated by electrophoresis under constant voltage, with the proteins being transferred onto PVDF membranes. The membranes are blocked at room temperature for 1 h and incubated overnight with the primary antibody at 4 °C. After washing the membranes three times with TBST, they are incubated with the secondary antibody for 1 h. Following three additional TBST washes, the blots are visualized using enhanced chemiluminescence. The anti-ser139-H2AX antibody is obtained from Cell Signaling Technology (rabbit, monoclonal, diluted 1:1000, Danvers, MA, USA). The anti-β-actin antibody is purchased from Beyotime (mouse, monoclonal, dilution 1:1000, Shanghai, China).

### 2.11. Intracellular Reactive Oxygen Species Detection

The generation of intracellular reactive oxygen species (ROS) was detected using a cell-permeable dye, 2′,7′-dichlorodihydrofluorescein diacetate (DCFH-DA), which reflects the formation of ROS within cells. The U87 cells were seeded into a 96-well plate at a density of 1 × 10^4^ cells per well and cultured for 24 h in a 37 °C incubator. Subsequently, wells with added materials served as the experimental group, while wells without any materials acted as the control group. After incubating for 4 h, the suspension was removed, and the cells were incubated with DCFH-DA in serum-free medium for 20 min. The cells were then washed with PBS, and the DCFH-DA buffer was replaced with a medium containing 10% FBS. Finally, the culture medium was removed, and the cell culture dishes were exposed to X-ray irradiation (4 Gy). Fluorescence was measured using a fluorometer (excitation at 488 nm/emission at 525 nm).

### 2.12. Extracellular Reactive Oxygen Species Detection

Aminophenyl fluorescein (APF) was utilized to detect the production of reactive oxygen species (ROS) by various synthesized nanoparticles under X-ray irradiation. Nanoparticles dissolved in ultrapure water (at a concentration of 20 μM) were mixed with 10 μM of prepared APF solution. This mixture was placed in 4 mL centrifuge tubes and shielded from light. The samples were then irradiated with X-rays at doses of 0, 2, 4, and 8 Gy (from a small animal irradiator, RS-2000, USA, at a dose rate of 160 kV, 25 mA, and 120 cGy/min). Subsequently, fluorescence values were measured using a fluorescence spectrophotometer (RF-6000, Shimadzu, Kyoto, Japan) with excitation at 490 nm and emission at 515 nm.

### 2.13. Statistical Analysis

Statistical analyses were conducted using SPSS 17.0 software. Comparisons between groups were performed through one-way ANOVA for independent samples. A *p*-value of less than 0.05 was considered statistically significant.

## 3. Results

### 3.1. Synthesis and Characterization of Rare-Earth Nanomaterials NaTm_x_Lu_(1−x)_F_4_

**Figure 2:**
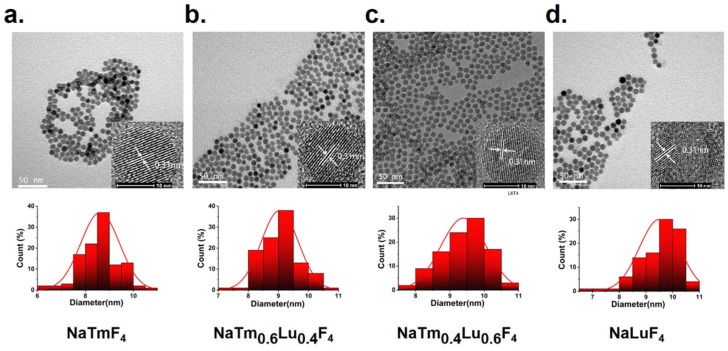
TEM and size distribution of four different rare-earth fluorides: (**a**) NaTmF_4_, (**b**) NaTm_0.6_Lu_0.4_F_4_, (**c**) NaTm_0.4_Lu_0.6_F_4_, (**d**) NaLuF_4_.

**Figure 3:**
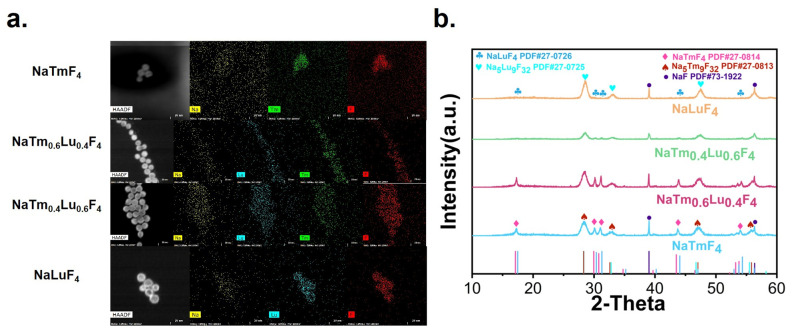
(**a**) EDS mapping of elements and (**b**) XRD pattern of as-synthesized four different nanoparticles.

Four types of nanoparticles, NaTm_x_Lu_(1−x)_F_4_ (x = 1, 0.6, 0.4, and 0, respectively), were synthesized using a hydrothermal method. Transmission electron microscopy results showed that these nanoparticles were spherical, with an approximate diameter of 9 nm and a consistent lattice spacing of 0.3 nm (Figure 2). This uniformity indicates that the size, shape, and lattice structure of all four types of nanoparticles are consistent. Energy-dispersive X-ray spectroscopy (EDS) confirmed the presence of Na, Tm, Lu, and F elements in the corresponding rare-earth nanoparticles (Figure 3a). X-ray powder diffraction was used to characterize the four nanoparticles, revealing that they consisted of hexagonal and tetragonal crystal structures. Specifically, the peaks were identified as mainly cubic phase NaTmF_4_ and NaLuF_4_, and the peaks of NaTm_0.6_Lu_0.4_F_4_ and NaTm_0.4_Lu_0.6_F_4_ were slightly shifted between the undoped pure phases. (Figure 3b). These nanoparticle sensitizers were then coated with a thin layer of silica through covalent bonding. Electron microscopy showed that the resulting water-soluble products (NaTmF_4_@SiO_2_, NaTm_0.6_Lu_0.4_F_4_@SiO_2_, NaTm_0.4_Lu_0.6_F_4_@SiO_2_, and NaLuF_4_@SiO_2_) had an approximate diameter of 10 nm, indicating that the silica coating was about 1–2 nm thick.

### 3.2. X-Ray Absorption Properties of the Nanoparticles

**Figure 4:**
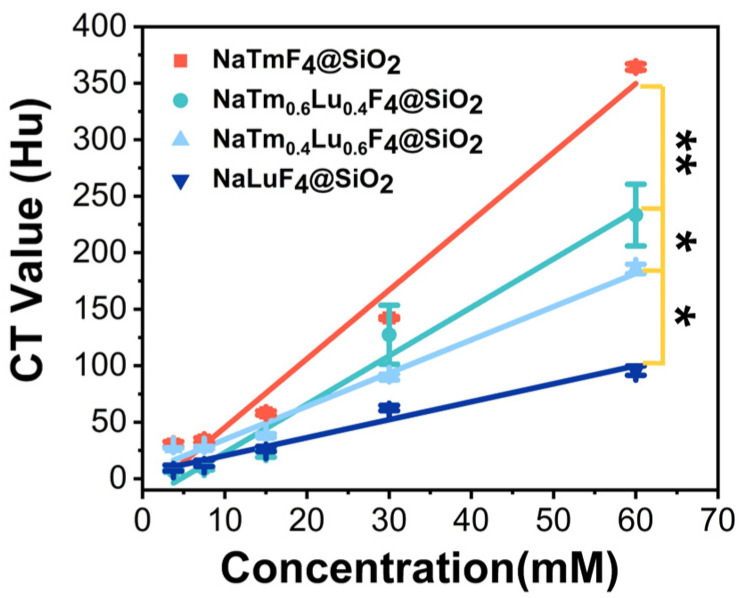
HU values of four different nanoparticles. *p* < 0.05 *, *p* < 0.01 **.

We then employed the computed tomography (CT) technique to demonstrate the X-ray attenuation of the nanoparticles because a CT uses an X-ray tube with voltages of 80~140 kV as the light source and a Bremsstrahlung peak exists at 59 keV. Figure 4 shows the variation curves of the Hounsfield Unit (HU) values for these four types of nanoparticles NaTm_x_Lu_(1−x)_F_4_ (where x = 1, 0.6, 0.4, 0, respectively) as a function of their concentration. The results demonstrate a linear increase in HU values for all nanomaterials with an increasing concentration. Additionally, an increase in the thulium (Tm) content in the nanoparticles correlates with a rising trend in HU values, with NaTmF_4_ nanoparticles exhibiting the highest attenuation slope. These findings demonstrate that nanoparticles containing Tm have a stronger X-ray radiation absorption capability than those with lutetium (Lu), although Tm has a smaller Z. Therefore, it can be inferred that the K-edge effect contributes to the higher X-ray attenuation of the nanoparticles containing Tm compared to those containing Lu. This result may also provide a clue for the design of CT contrast agents.

### 3.3. Secondary Electron Yield of Rare-Earth Nanomaterials

**Figure 5:**
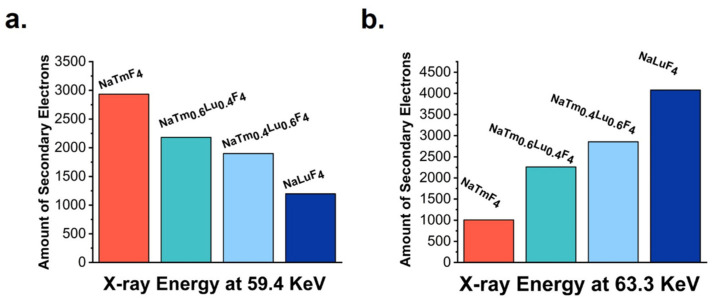
Using MCNP to calculate the secondary electrons generated by four different nanoparticles under the X-ray energies of 59.4 keV (**a**) and 63.3 keV (**b**).

The attenuation of X-rays by nanoparticles might be related to the number of secondary electrons they generate. To analyze this relationship, a Monte Carlo N Particle Transport Code System (MCNP) simulation method was employed to calculate the secondary electrons produced by four types of nanoparticles, as depicted in Figure 5. At a photon energy of 63.3 keV, corresponding to the K-edge binding energy of lutetium (Lu), NaLuF_4_ generated the highest number of secondary electrons, approximately four times that of NaTmF_4_. Conversely, as shown in Figure 5a, at a photon energy of 59.4 keV, NaTmF_4_ produced the most secondary electrons (2934), about 2.5 times more than NaLuF_4_. These results indicate that the maximum number of secondary electrons is produced when the X-ray irradiation energy is close to the atomic orbital binding energy of the irradiated element in the nanomaterial, rather than the higher atomic number.

### 3.4. Generation of Reactive Oxygen Species (ROS) by Rare-Earth Nanomaterials under X-Ray Irradiation

**Figure 6:**
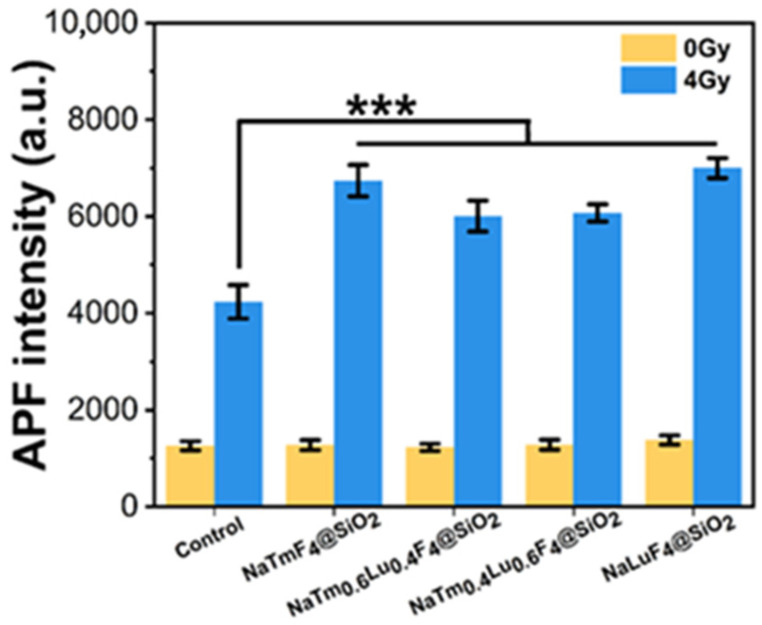
Generation of ROS of four different nanomaterials after irradiation by X-ray (dose: 0, 4 Gy), with deionized water as control group. *p* < 0.001 ***.

We further employed amino phenyl fluorescein (APF) as an indicator for detecting reactive oxygen species (ROS) generated by X-ray irradiation, as presented in Figure 6. Applying X-ray (4 Gy) enhanced the APF fluorescence intensity for all groups compared to that without irradiation (0 Gy), indicating that the irradiation caused ROS generation even without nanoparticles. Meanwhile, the more remarkable enhancement with the four nanoparticles suggests that part of the attenuated X-ray energy was emitted as ROS. Notably, there was no significant difference in the amount of ROS produced among the four types of nanoparticles under the same X-ray irradiation dose. This suggests that the generation of ROS by these rare-earth nanomaterials might not depend on the K-edge effect. Thus, the sensitization in lanthanide nanoparticle-based radiotherapy might involve the combined action of secondary electrons and reactive oxygen species.

### 3.5. Cytotoxicity and Cellular Phagocytosis Experiments with Rare-Earth Nanomaterials

**Figure 7:**
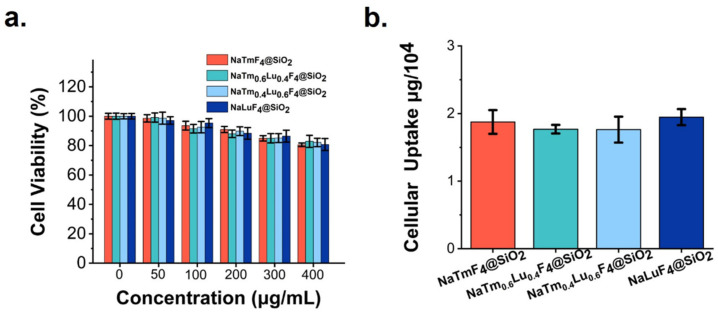
(**a**) Cell viability of glioma cell line U87 after incubating with different concentrations (0, 50 μg/mL, 100 μg/mL, 200 μg/mL, 300 μg/mL, and 400 μg/mL) of four different nanoparticles for 24 h. (**b**) Cellular uptake of different modified nanoparticles when treated with U87 cells for 4 h.

The cytotoxicity of four types of rare-earth nanomaterials was assessed using the CCK-8 assay. Glioblastoma U87 cells were co-cultured for 24 h with various concentrations (0, 50 μg/mL, 100 μg/mL, 200 μg/mL, 300 μg/mL, and 400 μg/mL) of these nanomaterials (NaTm_x_Lu_(1−x)_F_4_@SiO_2_, where x = 1, 0.6, 0.4, 0). Cell viability was then measured. As shown in Figure 7a, the survival rate of the U87 cells remained above 80% across all tested concentration ranges, indicating that all four types of rare-earth nanomaterials exhibit good cellular safety. Consequently, the subsequent experiments employed nanoparticle concentrations below 50 μg/mL to ensure a cell survival rate of over 90%. The results of the cellular uptake experiment are presented in Figure 7b. After incubating the U87 cells for 4 h with the four types of rare-earth nanomaterials at a concentration of 50 μg/mL, the measured uptake results were as follows: NaTmF_4_@SiO_2_ (1.8749 μg/10^4^ cells), NaTm_0.6_Lu_0.4_F_4_@SiO_2_ (1.768 μg/10^4^ cells), NaTm_0.4_Lu_0.6_F_4_@SiO_2_ (1.7619 μg/10^4^ cells), and NaLuF_4_@SiO_2_ (1.9468 μg/10^4^ cells). These findings indicate no significant difference in cellular uptake among the four types of rare-earth nanomaterials.

### 3.6. In Vitro Radiosensitization Experiments with Rare-Earth Nanomaterials

**Figure 8:**
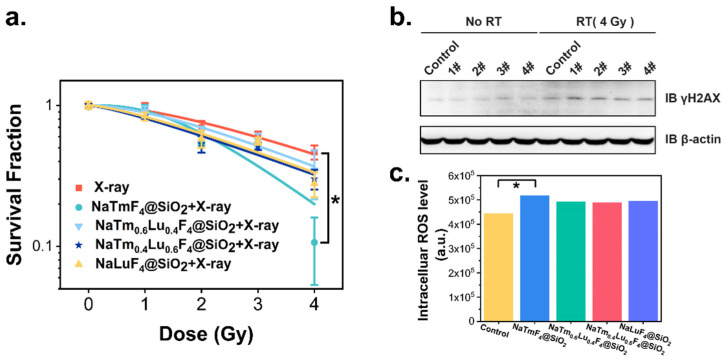
(**a**) The graph of cell viability and different X-ray doses of the four nanoparticles. (**b**) Western blot images of γ-H2AX with or without 4 Gy irradiation. (**c**) Intracellular ROS generation in U87 cells after treatment with the four nanoparticles under X-ray irradiation. *p* < 0.05 *. 1#: NaTmF_4_@SiO_2_, 2#: NaTm_0.6_Lu_0.4_F_4_@SiO_2_, 3#: NaTm_0.4_Lu_0.6_F_4_@SiO_2_, 4#: NaLuF_4_@SiO_2_.

Then, a cell colony formation assay was used to evaluate the radiosensitization effect on tumor cells. The U87 cells were cultured for 4 h with the four nanoparticles at a concentration of 10 μg/mL. Subsequently, the cells were irradiated with X-ray doses of 0, 1 Gy, 2 Gy, 3 Gy, and 4 Gy, respectively. After an additional two weeks of culture, the survival fraction (SF) of the U87 cells was calculated. As shown in Figure 8a, after 4 Gy X-ray irradiation, the survival rates of the U87 cells treated with NaTmF_4_@SiO_2_, NaTm_0.6_Lu_0.4_F_4_@SiO_2_, NaTm_0.4_Lu_0.6_F_4_@SiO_2_, and NaLuF_4_@SiO_2_ were 0.11, 0.35, 0.30, and 0.28, respectively, compared to 0.47 in the control group. This indicates an enhanced radiosensitization effect of NaTmF_4_@SiO_2_ at 4 Gy (*p* < 0.05). The sensitization enhancement ratios (SERs) at D0 and D10 were calculated by incorporating cell survival rates at different X-ray doses into the model. The obtained SERs for NaTmF_4_@SiO_2_, NaTm_0.6_Lu_0.4_F_4_@SiO_2_, NaTm_0.4_Lu_0.6_F_4_@SiO_2_, and NaLuF_4_@SiO_2_ were 2.06, 1.14, 1.01, and 1.05 at D0 and were 1.73, 1.16, 1.18, and 1.18 at D10, respectively, indicating a higher radiosensitization effect of NaTmF_4_@SiO_2_ than the other three nanoparticles. D0 represents the mean lethal dose corresponding to a 37% cell survival rate under X-ray irradiation, D10 is the dose for 10% cell survival, SER D0 is the sensitization ratio at 37% survival, and SERD10 is the sensitization ratio at 10% survival.

γH2AX, a member of the H2A histone family, serves as a biomarker reflecting DNA damage and repair, indicated by its phosphorylated form. As depicted in Figure 8b, after 4 Gy of radiotherapy, the four nanoparticles exhibited γH2AX expression, with NaTmF_4_@SiO_2_ showing a notably higher expression. This suggests that after 4 Gy of X-ray irradiation, the four nanoparticles enhanced DNA double-strand breaks in U87 cells, with NaTmF_4_@SiO_2_ causing slightly more DNA damage, possibly related to its higher generation of secondary electrons. The intracellular levels of reactive oxygen species (ROS) were also measured, as shown in Figure 8c. The ROS levels in U87 cells treated with the four nanoparticles were slightly higher than in the control group, with NaTmF_4_@SiO_2_ showing a marginally significant difference (*p* < 0.05). However, there was no significant difference in the ROS levels among the four nanoparticles within the U87 cells, consistent with the results shown in Figure 6, which indicated no significant difference in ROS production under X-ray irradiation.

## 4. Discussion

This study aimed to investigate the impact of the K-edge effect on the radiosensitization efficacy of rare-earth nanoparticles. Based on the current clinical use of X-ray sources with a Bremsstrahlung peak at 59 keV [38,39], thulium (Tm, Z = 69) was selected due to its K-edge energy of 59.4 keV, which is very close to this peak [40]. The four rare-earth nanoparticles, with different K-edge binding energies but identical diameters, lattices, and morphologies, were synthesized by adjusting the thulium content. Lutetium (Lu, Z = 71) with a higher atomic number but a different K-edge (63.3 keV) was used as a control [41]. CT characterization (HU values) revealed that the total attenuation of X-rays at the kilovolt (kV) level increased with the thulium content [42,43], consistent with the number of secondary electrons generated at the 59.4 keV X-ray photon energy. This shows that a higher Tm ratio produces more secondary electrons due to the K-edge effect, with NaTmF_4_ producing the most secondary electrons. Conversely, at an X-ray photon energy close to 63.3 keV, the order of secondary electron production was reversed. The experimental results, which contradict the classical mass attenuation coefficient theory by revealing that the production of secondary electrons is directly proportional to the fourth power of the atomic number, indicate that the K-edge effect has a more significant influence on secondary electron production than the atomic number. In vitro radiosensitization experiments showed that NaTmF_4_@SiO_2_ had a slightly higher radiosensitization effect, while the other three nanoparticle groups exhibited similar radiosensitization effects on the U87 cells. Furthermore, γ-H2AX protein blotting revealed that NaTmF_4_ induced slightly more DNA double-strand breaks in the U87 tumor cells, while the other three rare-earth nanoparticles did not show significant differences in DNA damage. Previous studies have demonstrated that secondary electrons generated by the interaction between X-rays and tantalum materials effectively induce DNA damage [44,45,46]. This suggests that the high secondary electron yield of NaTmF_4_@SiO_2_ is related to its radiosensitization mechanism. The results of this study highlight that the K-edge effect and secondary electron production contribute to enhancing the radiation sensitivity of rare-earth nanoparticles, providing a new direction for designing efficient nanoparticle radiosensitizers.

The generation of reactive oxygen species (ROS) is also a critical mechanism for enhancing the radiosensitization effect [47,48,49]. Carter et al. demonstrated through theoretical calculations and experiments that ROS radicals generated by gold nanoparticles under X-ray irradiation could induce DNA damage [50]. Therefore, in this study, we evaluated the ROS production by these nanoparticles under X-ray exposure and analyzed the impact of ROS on radiosensitization through clonogenic assays. The results showed that under 4 Gy of X-ray irradiation, there was no significant difference in ROS production among the four rare-earth nanoparticles. Within the X-ray dose range used in the clonogenic assay, NaTmF_4_@SiO_2_ exhibited the highest radiosensitization rate, with slightly higher ROS production in the U87 cells than the other three materials, possibly related to the increased secondary electron production. The survival rates of the U87 cells treated with the other three nanoparticles showed no significant differences, nor did the levels of ROS generated in the U87 cells. These findings suggest that there is no correlation between the amount of secondary electrons produced by the four nanoparticles and the levels of ROS generated. Based on the above results, it can be inferred that ROS is also an essential component in the radiosensitization mechanism of rare-earth nanoparticles. However, this study has some limitations, such as the lack of investigation into the intracellular distribution of nanoparticles after cellular uptake [7]. Understanding the distribution of nanoparticles within cellular organelles would help explain the primary pathways of ROS production and provide insights into the cellular uptake and clearance of these materials [51,52], which is crucial for assessing the biocompatibility and long-term effects of nanoparticles [47,53]. Our previous research has shown that NaGdF_4_:Yb, Er (UCNs) can inherently enhance the efficacy of radiotherapy, a process influenced by both cellular uptake and intracellular targeting, further demonstrating the significant role of ROS in the mechanism of radiosensitization [7].

Based on the conclusions of this study, the K-edge effect and the production of secondary electrons play a crucial role in enhancing radiosensitization. While the present study focuses on the radiosensitization effects of high-Z nanomaterials under kilovoltage (kV) X-ray irradiation, which is relevant for specific clinical applications such as intraoperative or superficial cancer treatments, the use of megavoltage (MV) photon beams (4–25 MeV) is more common in conventional teletherapy due to their better depth dose distribution [54,55,56]. At MV energies, the dominant interaction shifts from the photoelectric effect to Compton scattering, potentially reducing the radiosensitization efficiency of high-Z materials. Further studies are required to evaluate these effects systematically and determine the clinical applicability of high-Z nanomaterials under MV irradiation. Our findings also suggest that ROS may serve as an auxiliary mechanism in radiosensitization. Future research could explore other mechanisms by which rare-earth nanoparticles enhance radiation sensitivity. For example, their impact on cellular repair pathways, apoptosis, and autophagy could be significant in the radiosensitization process [57,58,59,60]. Additionally, investigating the interactions between rare-earth nanoparticles and various cellular components and their effects on cellular signaling pathways could provide a deeper understanding of the mechanisms underlying radiosensitization.

This study focused on the radiosensitization effects of Tm and Lu. Future research could compare the efficacy and mechanisms of nanoparticles with different compositions to gain further insights into the mechanisms of radiosensitization. Such comparative studies would help determine whether the observed effects are specific to Tm and Lu or represent the general characteristics of certain types of nanoparticles. Advanced imaging and analytical techniques, such as high-resolution electron microscopy, advanced spectroscopy, and single-cell sequencing, could be utilized in future research. These technologies can offer detailed information about these nanoparticles’ intracellular distribution, interactions with cellular structures, and impact on gene expression and protein function. The biocompatibility of the rare-earth nanoparticles used in this study was significantly improved by the 2 nm silica coating, as demonstrated by the reduced cytotoxicity observed in the U87 cells. While biodegradability and opsonization were not explicitly evaluated, the silica coating is expected to enhance colloidal stability and reduce protein adsorption, thereby improving nanoparticle transport efficiency. Future studies will focus on evaluating the long-term biodegradability and systemic behavior of these nanoparticles to fully assess their suitability for biological applications. The clinical application of rare-earth nanoparticles also requires further investigation to address issues related to biocompatibility, targeting efficiency, and long-term effects [61]. Ensuring the safety of these nanoparticles is crucial, and modifications may be necessary to enhance their targeting specificity to tumor cells while minimizing side effects in patients. In summary, this study reveals the significant impact of the K-edge effect on radiosensitization, providing valuable insights for the design of future nanoparticle radiosensitizers. Further in-depth research will help optimize the radiosensitization effects of rare-earth nanoparticles, ensuring their efficacy and safety in clinical applications.

## Data Availability

The original contributions presented in the study are included in the article, further inquiries can be directed to the corresponding authors.

## References

[1] Delaney G., Jacob S., Featherstone C., Barton M. (2005). The role of radiotherapy in cancer treatment: Estimating optimal utilization from a review of evidence-based clinical guidelines. Cancer.

[2] Baumann M., Krause M., Overgaard J., Debus J., Bentzen S.M., Daartz J., Richter C., Zips D., Bortfeld T. (2016). Radiation oncology in the era of precision medicine. Nat. Rev. Cancer.

[3] Haume K., Rosa S., Grellet S., Smialek M.A., Butterworth K.T., Solov’yov A.V., Prise K.M., Golding J., Mason N.J. (2016). Gold nanoparticles for cancer radiotherapy: A review. Cancer Nanotechnol..

[4] Wang Y., Liang R., Fang F. (2015). Applications of Nanomaterials in Radiotherapy for Malignant Tumors. J. Nanosci. Nanotechnol..

[5] Wang Y., Zhang H., Liu Y., Younis M.H., Cai W., Bu W. (2022). Catalytic radiosensitization: Insights from materials physicochemistry. Mater Today.

[6] Chen X., Liu J., Li Y., Pandey N.K., Chen T., Wang L., Amador E.H., Chen W., Liu F., Xiao E. (2022). Study of copper-cysteamine based X-ray induced photodynamic therapy and its effects on cancer cell proliferation and migration in a clinical mimic setting. Bioact. Mater..

[7] Xue J., Duosiken D., Zhong S., Cao J.J., Hu L.Y., Sun K., Tao K., Pan S.J. (2021). The dependence of radio-sensitization efficiency on mitochondrial targeting with NaGdF(4):Yb,Er nanoparticles. Acta Biomater..

[8] Zhang C., Yan L., Gu Z., Zhao Y. (2019). Strategies based on metal-based nanoparticles for hypoxic-tumor radiotherapy. Chem. Sci..

[9] Liu Y., Zhang P., Li F., Jin X., Li J., Chen W., Li Q. (2018). Metal-based NanoEnhancers for Future Radiotherapy: Radiosensitizing and Synergistic Effects on Tumor Cells. Theranostics.

[10] Garcia-Prada C.D., Carmes L., Atis S., Parach A., Bertolet A., Jarlier M., Poty S., Garcia D.S., Shin W.G., Du Manoir S. (2023). Gadolinium-Based Nanoparticles Sensitize Ovarian Peritoneal Carcinomatosis to Targeted Radionuclide Therapy. J. Nucl. Med..

[11] Ma X., Liang X., Yao M., Gao Y., Luo Q., Li X., Yu Y., Sun Y., Cheng M.H.Y., Chen J. (2023). Myoglobin-loaded gadolinium nanotexaphyrins for oxygen synergy and imaging-guided radiosensitization therapy. Nat. Commun..

[12] Maiti D., Yu H., Mochida Y., Won S., Yamashita S., Naito M., Miyata K., Kim H.J. (2023). Terbium-Rose Bengal Coordination Nanocrystals-Induced ROS Production under Low-Dose X-rays in Cultured Cancer Cells for Photodynamic Therapy. ACS Appl. Bio Mater..

[13] Moradi M.S., Bidabadi B.S. (2020). Assessment of Single-and Double-Strand Breaks in DNA Induced by Auger Electrons of Radioisotopes Used in Diagnostic and Therapeutic Applications. J. Med. Phys..

[14] Simpkin D.J. (1999). Radiation interactions and internal dosimetry in nuclear medicine. Radiographics.

[15] Jayarathna S., Kaphle A., Krishnan S., Cho S.H. (2024). Nanoscale gold nanoparticle (GNP)-laden tumor cell model and its use for estimation of intracellular dose from GNP-induced secondary electrons. Med. Phys..

[16] Dai Q., Wang L., Ren E., Chen H., Gao X., Cheng H., An Y., Chu C., Liu G. (2022). Ruthenium-Based Metal-Organic Nanoradiosensitizers Enhance Radiotherapy by Combining ROS Generation and CO Gas Release. Angew. Chem. Int. Ed. Engl..

[17] Soesbe T.C., Xi Y., Nasr K., Leyendecker J.R., Lenkinski R.E., Lewis M.A. (2021). Investigating new CT contrast agents: A phantom study exploring quantification and differentiation methods for high-Z elements using dual-energy CT. Eur. Radiol..

[18] Lin C.F., Huang K.W., Chen Y.T., Hsueh S.L., Li M.H., Chen P. (2023). Perovskite-Based X-ray Detectors. Nanomaterials.

[19] Schwartz-Duval A.S., Mackeyev Y., Mahmud I., Lorenzi P.L., Gagea M., Krishnan S., Sokolov K.V. (2024). Intratumoral Biosynthesis of Gold Nanoclusters by Pancreatic Cancer to Overcome Delivery Barriers to Radiosensitization. ACS Nano.

[20] Li J., Lv Z., Guo Y., Fang J., Wang A., Feng Y., Zhang Y., Zhu J., Zhao Z., Cheng X. (2023). Hafnium (Hf)-Chelating Porphyrin-Decorated Gold Nanosensitizers for Enhanced Radio-Radiodynamic Therapy of Colon Carcinoma. ACS Nano.

[21] Hua Y., Shao Z.H., Zhai A., Zhang L.J., Wang Z.Y., Zhao G., Xie F., Liu J.Q., Zhao X., Chen X. (2023). Water-Soluble Au(25) Clusters with Single-Crystal Structure for Mitochondria-Targeting Radioimmunotherapy. ACS Nano.

[22] Li J., Xie L., Sang W., Li W., Wang G., Yan J., Zhang Z., Tian H., Fan Q., Dai Y. (2022). A Metal-Phenolic Nanosensitizer Performs Hydrogen Sulfide-Reprogrammed Oxygen Metabolism for Cancer Radiotherapy Intensification and Immunogenicity. Angew. Chem. Int. Ed. Engl..

[23] Jiang Z., Zhang M., Li P., Wang Y., Fu Q. (2023). Nanomaterial-based CT contrast agents and their applications in image-guided therapy. Theranostics.

[24] Kubiak T. (2017). Nanoparticles as radiosensitizers in photon and hadron radiotherapy. Acta Bio-Opt. Inf. Med. Biomed. Eng..

[25] Turnbull T., Douglass M., Williamson N.H., Howard D., Bhardwaj R., Lawrence M., Paterson D.J., Bezak E., Thierry B., Kempson I.M. (2019). Cross-Correlative Single-Cell Analysis Reveals Biological Mechanisms of Nanoparticle Radiosensitization. ACS Nano.

[26] Grall R., Girard H., Saad L., Petit T., Gesset C., Combis-Schlumberger M., Paget V., Delic J., Arnault J.C., Chevillard S. (2015). Impairing the radioresistance of cancer cells by hydrogenated nanodiamonds. Biomaterials.

[27] Rosa S., Connolly C., Schettino G., Butterworth K.T., Prise K.M. (2017). Biological mechanisms of gold nanoparticle radiosensitization. Cancer Nanotechnol..

[28] Jain S., Coulter J.A., Hounsell A.R., Butterworth K.T., McMahon S.J., Hyland W.B., Muir M.F., Dickson G.R., Prise K.M., Currell F.J. (2011). Cell-specific radiosensitization by gold nanoparticles at megavoltage radiation energies. Int. J. Radiat. Oncol. Biol. Phys..

[29] Lin Y., McMahon S.J., Scarpelli M., Paganetti H., Schuemann J. (2014). Comparing gold nano-particle enhanced radiotherapy with protons, megavoltage photons and kilovoltage photons: A Monte Carlo simulation. Phys. Med. Biol..

[30] Coursey C.A., Nelson R.C., Boll D.T., Paulson E.K., Ho L.M., Neville A.M., Marin D., Gupta R.T., Schindera S.T. (2010). Dual-energy multidetector CT: How does it work, what can it tell us, and when can we use it in abdominopelvic imaging?. Radiographics.

[31] Higashi Y., Matsumoto K., Saitoh H., Shiro A., Ma Y., Laird M., Chinnathambi S., Birault A., Doan T.L.H., Yasuda R. (2021). Iodine containing porous organosilica nanoparticles trigger tumor spheroids destruction upon monochromatic X-ray irradiation: DNA breaks and K-edge energy X-ray. Sci. Rep..

[32] Biston M.C., Joubert A., Adam J.F., Elleaume H., Bohic S., Charvet A.M., Esteve F., Foray N., Balosso J. (2004). Cure of Fisher rats bearing radioresistant F98 glioma treated with cis-platinum and irradiated with monochromatic synchrotron X-rays. Cancer Res..

[33] Yokoya A., Cunniffe S.M., Watanabe R., Kobayashi K., O’Neill P. (2009). Induction of DNA strand breaks, base lesions and clustered damage sites in hydrated plasmid DNA films by ultrasoft X rays around the phosphorus K edge. Radiat. Res..

[34] Nan W., Zhang L., Li S., Jin P., Zhou B., Yin Z., Wang T., Ye N., Hu Z., Wu Y. (2024). Achieving Ultrahigh Photoluminescence Quantum Yield in Highly Stable Cs(3)Cu(2)I(5) Perovskite Single Crystals Through Melt Growth. Inorg. Chem..

[35] Ballester F., Granero D., Perez-Calatayud J., Venselaar J.L., Rivard M.J. (2010). Study of encapsulated 170Tm sources for their potential use in brachytherapy. Med. Phys..

[36] Liu Y., Li X., Yin Y., Li Z., Yao H., Li Z., Li H. (2024). Design and Computational Validation of gamma-Ray Shielding Effectiveness in Heavy Metal/Rare Earth Oxide-Natural Rubber Composites. Polymers.

[37] Liu Q., Sun Y., Yang T., Feng W., Li C., Li F. (2011). Sub-10 nm hexagonal lanthanide-doped NaLuF4 upconversion nanocrystals for sensitive bioimaging in vivo. J. Am. Chem. Soc..

[38] Bartkoski D.A., Bar-David A., Kleckner M., Mirkovic D., Tailor R., Moradi-Kurdestany J., Borukhin S., Harel Z., Burshtein Z., Zuck A. (2021). Analysis of a novel X-ray lens for converging beam radiotherapy. Sci. Rep..

[39] Pomme S., Paepen J., Marouli M. (2019). Conversion electron spectroscopy of the 59.54 keV transition in (241)Am alpha decay. Appl. Radiat. Isot..

[40] Gonzalez-Mancebo D., Becerro A.I., Caro C., Gomez-Gonzalez E., Garcia-Martin M.L., Ocana M. (2024). Nanoparticulated Bimodal Contrast Agent for Ultra-High-Field Magnetic Resonance Imaging and Spectral X-ray Computed Tomography. Inorg. Chem..

[41] Zou Y., Fischer C.F. (2002). Resonance transition energies and oscillator strengths in lutetium and lawrencium. Phys. Rev. Lett..

[42] Taylor P.A., Mirandola A., Ciocca M., Hartzell S., Vai A., Alvarez P., Howell R.M., Koay E.J., Peeler C.R., Peterson C.B. (2024). Technical note: Radiological clinical equivalence for phantom materials in carbon ion therapy. Med. Phys..

[43] Boussoussou M., Vattay B., Szilveszter B., Simon J., Lin A., Vecsey-Nagy M., Konkoly G., Merkely B., Maurovich-Horvat P., Dey D. (2023). The effect of patient and imaging characteristics on coronary CT angiography assessed pericoronary adipose tissue attenuation and gradient. J. Cardiovasc. Comput. Tomogr..

[44] Cai Z., Cloutier P., Hunting D., Sanche L. (2006). Enhanced DNA damage induced by secondary electron emission from a tantalum surface exposed to soft x rays. Radiat. Res..

[45] McElligott O., Nikandrovs M., McCavana P., McClean B., Leon Vintro L. (2024). Estimation of the relative biological effectiveness for double strand break induction of clinical kilovoltage beams using Monte Carlo simulations. Med. Phys..

[46] Huwaidi A., Robert G., Kumari B., Bass A.D., Cloutier P., Guerin B., Sanche L., Wagner J.R. (2024). Electron-Induced Damage by UV Photolysis of DNA Attached to Gold Nanoparticles. Chem. Res. Toxicol..

[47] Slama Y., Arcambal A., Septembre-Malaterre A., Morel A.L., Pesnel S., Gasque P. (2024). Evaluation of core-shell Fe(3)O(4)@Au nanoparticles as radioenhancer in A549 cell lung cancer model. Heliyon.

[48] Mansouri E., Mesbahi A., Hamishehkar H., Montazersaheb S., Hosseini V., Rajabpour S. (2023). The effect of nanoparticle coating on biological, chemical and biophysical parameters influencing radiosensitization in nanoparticle-aided radiation therapy. BMC Chem..

[49] Xiao W., Zhao L., Sun Y., Yang X., Fu Q. (2024). Stimuli-Responsive Nanoradiosensitizers for Enhanced Cancer Radiotherapy. Small Methods.

[50] Carter J.D., Cheng N.N., Qu Y., Suarez G.D., Guo T. (2007). Nanoscale energy deposition by X-ray absorbing nanostructures. J. Phys. Chem. B.

[51] Asadi N., Gharbavi M., Rezaeejam H., Farajollahi A., Johari B. (2024). Zinc nanoparticles coated with doxorubicin-conjugated alginate as a radiation sensitizer in triple-negative breast cancer cells. Int. J. Pharm..

[52] Hu M., Bao J., Zhang Y., Wang L., Zhang Y., Zhang J., Tang J., Zou Q. (2024). Supramolecular Nanoparticles of Histone and Hyaluronic Acid for Co-Delivery of siRNA and Photosensitizer In Vitro. Int. J. Mol. Sci..

[53] Yang X., Wang X., Zhang X., Zhang J., Lam J.W.Y., Sun H., Yang J., Liang Y., Tang B.Z. (2024). Donor-Acceptor Modulating of Ionic AIE Photosensitizers for Enhanced ROS Generation and NIR-II Emission. Adv. Mater..

[54] Prior P., Awan M.J., Wilson J.F., Li X.A. (2021). Tumor Control Probability Modeling for Radiation Therapy of Keratinocyte Carcinoma. Front. Oncol..

[55] Khaledi N., Hayes C., Belshaw L., Grattan M., Khan R., Grafe J.L. (2022). Treatment planning with a 2.5 MV photon beam for radiation therapy. J. Appl. Clin. Med. Phys..

[56] Schmidt R.M., Hara D., Vega J.D., Abuhaija M.B., Tao W., Dogan N., Pollack A., Ford J.C., Shi J. (2022). Quantifying Radiosensitization of PSMA-Targeted Gold Nanoparticles on Prostate Cancer Cells at Megavoltage Radiation Energies by Monte Carlo Simulation and Local Effect Model. Pharmaceutics.

[57] Rachamala H.K., Madamsetty V.S., Angom R.S., Nakka N.M., Kumar Dutta S., Wang E., Mukhopadhyay D., Pal K. (2023). Targeting mTOR and Survivin Concurrently Potentiates Radiation Therapy in Renal Cell Carcinoma by Suppressing DNA Damage Repair and Amplifying Mitotic Catastrophe. Res. Sq..

[58] Melia E., Parsons J.L. (2023). DNA damage and repair dependencies of ionising radiation modalities. Biosci. Rep..

[59] Zhang Y.M., Miao Z.M., Chen Y.P., Song Z.B., Li Y.Y., Liu Z.W., Zhou G.C., Li J., Shi L.L., Chen Y. (2024). Ononin promotes radiosensitivity in lung cancer by inhibiting HIF-1alpha/VEGF pathway. Phytomedicine.

[60] Park M., Ha J., Lee Y., Kwon Y., Choi S.H., Kim B.S., Jeong Y.K. (2023). BR101801 enhances the radiosensitivity of p53-deficient colorectal cancer cells by inducing G2/M arrest, apoptosis, and senescence in a p53-independent manner. Am. J. Cancer Res..

[61] Farzin L., Sheibani S., Moassesi M.E., Shamsipur M. (2019). An overview of nanoscale radionuclides and radiolabeled nanomaterials commonly used for nuclear molecular imaging and therapeutic functions. J. Biomed. Mater. Res. A.

